# Serine 26 in the PomB Subunit of the Flagellar Motor Is Essential for Hypermotility of *Vibrio cholerae*

**DOI:** 10.1371/journal.pone.0123518

**Published:** 2015-04-15

**Authors:** Petra Halang, Thomas Vorburger, Julia Steuber

**Affiliations:** Institute of Microbiology, University of Hohenheim (Stuttgart), Stuttgart, Germany; Centre National de la Recherche Scientifique, Aix-Marseille Université, FRANCE.

## Abstract

*Vibrio cholerae* is motile by means of its single polar flagellum which is driven by the sodium-motive force. In the motor driving rotation of the flagellar filament, a stator complex consisting of subunits PomA and PomB converts the electrochemical sodium ion gradient into torque. Charged or polar residues within the membrane part of PomB could act as ligands for Na^+^, or stabilize a hydrogen bond network by interacting with water within the putative channel between PomA and PomB. By analyzing a large data set of individual tracks of swimming cells, we show that S26 located within the transmembrane helix of PomB is required to promote very fast swimming of *V*. *cholerae*. Loss of hypermotility was observed with the S26T variant of PomB at pH 7.0, but fast swimming was restored by decreasing the H^+^ concentration of the external medium. Our study identifies S26 as a second important residue besides D23 in the PomB channel. It is proposed that S26, together with D23 located in close proximity, is important to perturb the hydration shell of Na^+^ before its passage through a constriction within the stator channel.

## Introduction

**Fig 1:**
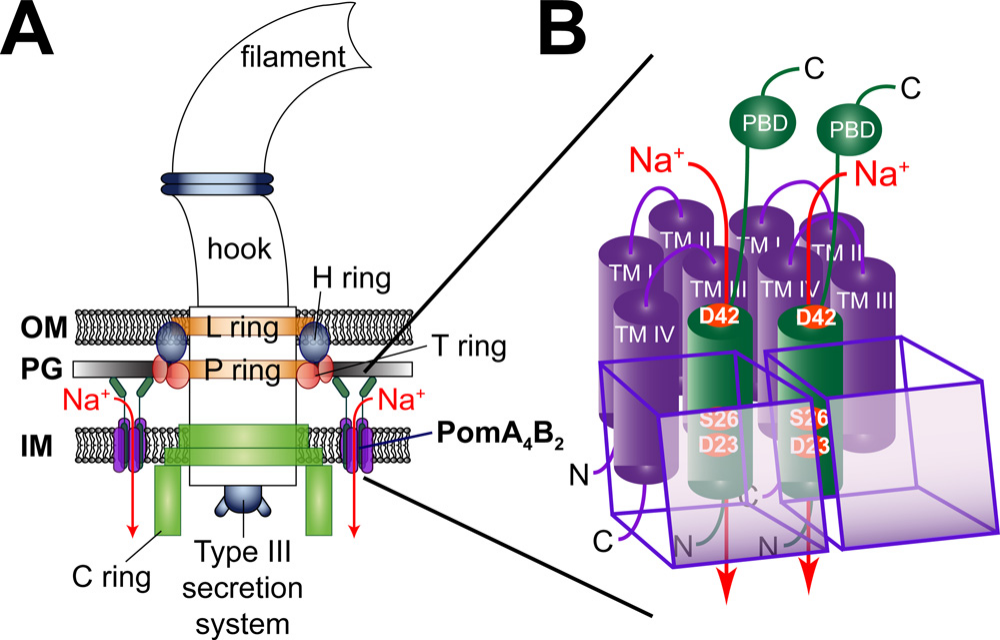
Rotation of the single polar flagellum from *V*. *cholerae* is driven by a Na^+^ flux through the PomA_4_PomB_2_ stator complex. A: The flagellum of *V*. *cholerae* consists of rotating and static parts. The filament, the hook, which connects the filament to the motor complex, the membrane embedded L and P rings in the outer membrane (OM), and the C ring in the inner membrane (IM) rotate against several stator complexes, each composed of four PomA and two PomB proteins. The H ring functions as a bushing to enable rotation against the peptidoglycan layer (PG). The T ring connects the stator complexes with the peptidoglycan layer. Proton-driven flagella, such as found in *E*. *coli* and *Salmonella*, do not have H and T rings. Export of protein components of the flagellar filament is catalyzed by a type III secretion system located at the base of the flagellum. B: Four PomA and two PomB subunits form the flagellar stator complex. PomA possesses four transmembrane helices (TM I–IV), PomB only one. The translucent, purple boxes in the front each represent one PomA subunit. The flux of Na^+^ through the stator complex (red arrow) along the chemical gradient drives the rotation of the C ring against the stator elements. It is assumed that Na^+^ ions pass through a channel built by helices III and IV of PomA and the single transmembrane helix of PomB [17,31]. The peptidoglycan binding domain (PBD) in the C-terminal part of PomB stabilizes the stator complexes within the peptidoglycan layer. The conserved amino acid residues D42, S26 and D23 of PomB promote the transport of Na^+^ through the stator [52].

It is essential for bacteria to be able to move, either away from a repellent or towards an attractive environment [1,2]. Motility is achieved via extracellular helical filaments—called flagella, which can be rotated clock- or counterclockwise [3]. The bacterial flagellum consists of three distinct parts: a hollow filament with a length of 15–20 μm composed of flagellin; the hook, which connects the filament to the motor complex; and the membrane embedded motor complex consisting of a rotor and a stator (Fig 1) (reviewed in [4,5]). Typically, the flagellum is driven either by a proton- or a sodium-motive force [6–9], but the mechanism converting ion flux into torque still remains unclear. The stator complex defines the specificity to either protons or sodium ions, with four MotA and two MotB subunits forming a H^+^-dependent MotA_4_MotB_2_ stator [10–14], and the homologous PomA and PomB subunits forming a Na^+^-dependent PomA_4_PomB_2_ stator [15–19] (Fig 1). Despite considerable effort [20–23], critical amino acid residue(s) conveying H^+^ or Na^+^ specificity in the stator could not yet be identified, reviewed by Sowa and Berry who stated that “there is no single determining component for ion selectivity” [24]. Most of the bacteria whose flagellar motors were studied to date use a single type of stator complex with a distinct selectivity towards a single coupling ion (H^+^ or Na^+^). However, there are exceptions to this rule. One is the metal ion-reducing bacterium *Shewanella oneidensis*. It is motile by means of a single polar flagellum whose motor is, depending on the Na^+^ concentration, equipped with either PomAB (Na^+^) or MotAB (H^+^) stators [25]. Another exception is the alkaliphilic *Bacillus clausii* that possesses a single, bifunctional stator which couples motility to Na^+^ at the high end of its pH range (pH 7–11), but uses protons at lower pH [26]. Pairs of mutations in *Bacillus clausii* MotB were identified that converted the naturally bifunctional MotAB stator complex into stators that preferentially use either H^+^ oder Na^+^ across the full pH range [26]. A dual use of Na^+^ and K^+^ as coupling ions for flagellar motors should also be considered [27].

*V*. *cholerae* possesses a single, polar flagellum whose Na^+^-driven motor is equipped with PomAB stator complexes [28,29]. PomA possesses four transmembrane helices and a large cytoplasmic loop between helix II and III, while PomB consists of one transmembrane helix and a large periplasmic domain (Fig 1) [29]. It was proposed that helices III and IV of the A subunit and the single transmembrane helix of the B subunit are in close proximity to form an ion channel [30–34]. The C terminal part of subunit B—which is essential for stator function—reaches into the periplasmic space and attaches itself to the peptidoglycan layer with its special peptidoglycan binding motif, which increases the stability of the stator complex in the cell membrane [35,36]. The number of stator complexes which surround the motor ranges between 11 and 12 [37,38]. With regard to the high rotational speeds (up to 1700 Hz) which were determined for the sodium-dependent polar flagellum of *V*. *alginolyticus*, it was first assumed that the rotor and stator complexes of the flagellar motor might form a tight and stable complex [39]. Nonetheless it could be demonstrated that stator complexes dynamically associate with or dissociate from the motor without disrupting flagellar rotation [37], in equilibrium with a pool of membrane embedded, inactive stator complexes [40,41]. It is assumed that upon ion influx, conformational changes occur in the cytoplasmic loop of PomA bringing the cytoplasmic loop in close proximity to FliG [42]. Charged residues of this cytoplasmic loop interact with charged residues residing in the C-terminal domain of FliG. FliG is part of the torque generating machinery and switch complex responsible for changing the direction of flagellar motor rotation. It also participates in the assembly of the flagellum and is important for efficient stator assembly around a rotor [22,43–51]. When searching for a putative ion pathway through the flagellar motor, one has to consider the topology and the position of charged amino acid residues in the membrane-embedded stretches of the stator. We recently showed that D23, S26, and D42 of PomB are part of ion-conducting pathway(s) in the PomA_4_B_2_ stator complex of the Na^+^-driven flagellum from *V*. *cholerae* [52]. In the present study, a large number of tracks of *V*. *cholerae* Δ*pomAB* cells expressing PomB-S26A or PomB-S26T variants were analyzed to study the influence of pH and salt on the distribution of swimming speeds. To this end, a novel microscopic approach was applied which did not require staining of cells with fluorophores. It has been suggested that the exchange of PomB-S26 (conserved in Na^+^-dependent flagellar motors) for a conserved threonine at the corresponding position in H^+^-dependent motors might reflect their different ion specificity [53]. Here we show that S26 is critical to promote very fast rotation of the flagellum of *V*. *cholerae*. We propose that S26 is important for the orientation of water molecules, and helps to perturb the hydration shell of Na^+^ before entering the selectivity filter of the channel in the stator.

## Materials and Methods

### Chemicals, bacterial strains and plasmids

Unless stated otherwise, chemicals were purchased from Sigma-Aldrich Chemie GmbH, Germany. Yeast extract, tryptone, and bacto agar were purchased from Becton, Dickinson. The *V*. *cholerae* strains used in this study were O395-N1 [54], O395-N1 Δ*pomAB* (Δ*ctxA* Str^r^ Δ*pomAB*) [22] and O395-N1 Δ*nqr* (Δ*ctxA* Str^r^ Δ*nqrA-F*) [55]. The plasmids used in this study are listed in Table 1.

**Table 1:** Plasmids used in this study.

Plasmid	Relevant characteristics	Reference
pAB	*P*_*araBAD*_, codes for His_6_-PomA and PomB-Strep, Ap^r^	[29,52]
pAB-S26A	*P*_*araBAD*_, codes for His_6_-PomA and S26A variant of PomB-Strep, Ap^r^	[52]
pAB-S26T	*P*_*araBAD*_, codes for His_6_-PomA and S26T variant of PomB-Strep, Ap^r^	[52]
pISC-H	Empty vector of pAB	[29]

### Isolation of total RNA from *V*. *cholerae*

5 ml LB-Na^+^ (1% tryptone, 0.5% yeast extract, 171 mM NaCl, 50 μg ml^-1^ streptomycin, pH 7.0) was inoculated with an overnight culture of either *V*. *cholerae* reference strain or *V*. *cholerae* Δ*pomAB* transformed with pAB to an OD_600_ of 0.01. Ampicillin (200 μg ml^-1^) and L-(+)-arabinose (10 mM) was added for *V*. *cholerae* Δ*pomAB* pAB. Cells were grown aerobically at 30°C / 180 rpm (orbit: 25 mm) until an OD_600_ of approximately 0.5 was reached, harvested by centrifugation (2 min, 11.000 rpm) and washed in 1 ml RNA*later* (Qiagen). Cells were harvested (2 min, 11.000 rpm) and resuspended in 0.5 ml TE buffer (30 mM Tris-HCl, 1 mM EDTA, pH 8.0) containing lysozyme (5 mg ml^-1^), CaCl_2_ (1 mM), MgCl_2_ (1 mM) and DNase I (a few crystals were dissolved in 10 ml of TE buffer). Cells were incubated at 30°C for 20 min prior to addition of proteinase K (1 mg ml^-1^). Incubation at 30°C was continued (15 min), and RNA was purified using the RNeasy Mini Kit (Qiagen) according to the manufacturer’s protocol. The quality of the isolated RNA was assessed by gel electrophoresis. RiboRuler High Range RNA Ladder (Thermo Scientific) was used as molecular standard. We did not detect bands beside the 23S and 16S rRNA, indicating the absence of genomic DNA. The RNA yield was 414 ng μl^-1^ from the *V*. *cholerae* reference strain and 130 ng μl^-1^ from the *pomAB* deletion strain transformed with pAB.

### Real-Time Quantitative Reverse Transcription PCR (qRT-PCR)

The SuperScript III First-Strand Synthesis System for RT-PCR (Invitrogen) was used for the synthesis of cDNA according to the manufacturer’s protocol using random hexamer primers. The yields of the cDNA preparations that served as template source for the qRT-PCR reactions were 166 ng μl^-1^ for the *V*. *cholerae* reference strain and 52 ng μl^-1^ for *V*. *cholerae* Δ*pomAB* pAB. The SensiFAST SYBR & Fluorescein Kit (Bioline) was used to determine the expression level of *pomB* in qRT-PCR reactions using primer pairs “pomB fwd” (5´-CGCAGTTTCGGTGGCGCAAG-3´) and “pomB rev” (5´-TGCCCGTTGCGCTTCGGTAT-3´). A reaction mix (20 μl) contained 20 pmol of each primer and 100 ng cDNA. qRT-PCR reactions were performed in triplicates using a CFX96 cycler (Bio-Rad) with following cycling conditions: 1.95°C for 2 min, 2.95°C for 10 s, 3.60°C for 20 s, 4.95°C for 10 s, and a final melting curve with a temperature range from 65°C to 95°C (0.5°C increment). Steps 2 and 3 were repeated 39 times. The correct size (134 bp) of the qRT-PCR products was confirmed by gel-electrophoresis using a 100 bp DNA ladder (GeneRuler, Thermo Scientific). Primer efficiencies (with 100 ng, 10 ng, 1 ng, 0.1 ng cDNA as template) and relative gene expression of *pomB* from the *V*. *cholerae* reference strain and *V*. *cholerae* Δ*pomAB* pAB were quantified using the CFX Manager software (Bio-Rad, version 2.1.1022.0523). The primer efficiency was 115% with R^2^ = 0.998.

### Detection of PomB variants on Western blots

5 ml LB-Na^+^ (1% tryptone, 0.5% yeast extract, 171 mM NaCl, 10 mM Tris base, 50 μg ml^-1^ streptomycin, 200 μg ml^-1^ ampicillin, pH adjusted to 8.0 with 5 M KOH) was inoculated to an OD_600_ of 0.01 with an overnight culture of *V*. *cholerae* Δ*pomAB* transformed with pISC-H, pAB, pAB-S26A or pAB-S26T, respectively. Overexpression of His_6_-PomA together with PomB-Strep (wild type), PomB-S26A-Strep or PomB-S26T-Strep was performed in the presence of 10 mM L-(+)-arabinose. After 4 h of growth at 30°C / 180 rpm (orbit: 25 mm), cells were harvested by centrifugation (15 min, 11.000 rpm) and resuspended in SDS loading buffer to a final OD_600_ of 5.0. Assuming that 1 U of absorbance at 600 nm corresponds to 0.33 g total dry weight per liter [56] and that 55% of the total dry weight represents protein [57], the 20 μl of the cell suspension in SDS loading buffer which were loaded onto the SDS-PAGE gel per lane contained 18 μg total protein. SDS-PAGE and Western blot analysis were performed as described in [29].

### Motility of *V*. *cholerae* on softagar plates

Motility assays were performed on LB softagar plates pH 8.0 as described in [58] with the following L-(+)-arabinose concentrations: 0, 0.001, 0.01, 0.1, 1.0, 10 and 20 mM. Plates were incubated at 30°C for 21 h. The average and standard deviations were calculated with eight experiments from a total of 10 experiments (the lowest and highest values were omitted).

### Determination of swimming speeds by microscopy

With *V*. *cholerae*, we did not observe any motile cells in the buffer used by Homma and coworkers to study motility of *V*. *alginolyticus* [53]. Instead, experiments were performed in LB-based tracking media (1% tryptone, 0.5% yeast extract, 10 mM Tris base, 50 μg ml^-1^ streptomycin) without added salts, or with 171 mM NaCl (LB-Na^+^) or 171 mM KCl (LB-K^+^). The pH was adjusted to 7.0, 8.0 or 9.0 with 5 M HCl or 5 M KOH. Media for *V*. *cholerae* Δ*pomAB* transformed with plasmids pAB, pAB-S26A or pAB-S26T contained 200 μg ml^-1^ ampicillin and 10 mM L-(+)-arabinose. The residual Na^+^ and K^+^ concentrations in the medium without added salts were 11 mM and 12 mM, as determined by atomic absorption spectroscopy (Shimadzu AA-6200). An overnight culture of a given *V*. *cholerae* strain grown in LB-Na^+^ at 37°C was diluted in LB-based tracking medium with indicated pH and salt content to an OD_600_ of 0.01. Cells were incubated at 30°C under shaking (180 rpm, orbit: 25 mm) for 4 h in media with salt added, or 5 h in media without added salts. Cells were diluted 1:100 in the same medium, and 50 mM L-serine was added to enhance straight swimming of the bacteria. An aliquot (30 μL) was pipetted into one channel of a flat μ-Slide VI (Ibidi, Germany), and cells were immediately observed under 200-fold magnification using an upright fluorescence microscope (Imager M1, Zeiss, Germany) in its differential interference contrast (DIC) mode. The microscope was equipped with a standard CCD camera (AxioCam MRm, Zeiss, Germany) and controlled using the software AxioVision (Zeiss, Germany; release 4.7) including a real-time video recorder function (Fast Acquisition Module, Zeiss, Germany). After determination of adequate settings for the light exposure using the auto-exposure function of AxioVision and manual focusing in the z-axis to the middle between the base and the top of the channel, about 30 series of time sequences were taken. The typical exposure time was 20 ms, and the constant interval between two consecutive images was 50 ms. The total length of a typical sequence of images was 12 s, corresponding to 250 individual images in one tracking experiment. At least 305 tracks per condition and strain were recorded and analyzed by the WimTaxis software (Wimasis GmbH, Germany). All bacterial cells showing a minimum speed of 4 μm s^-1^ were included in the analysis. Bacterial cells were divided into three main classes according to their measured swimming speeds, namely class I: from 4 μm s^-1^ to less than 18 μm s^-1^ (“slow swimmers”), class II: from 18 μm s^-1^ to less than 41 μm s^-1^ (“medium swimmers”), and class III: 41 μm s^-1^ and faster (“fast swimmers”). Results were presented in histograms created with the help of the software Origin (release 8.0). The average of velocities in a given class, the standard deviation, the mean and the median were calculated using Origin.

### Membrane potential (Δψ) measurements

Δψ established by *V*. *cholerae* cells was assayed using the BacLight Bacterial Membrane Potential Kit (Invitrogen) basically according to the manufacturer’s instructions. The staining dye 3,3´-diethyloxacarbocyanine iodide (DiOC_2_) exhibits green fluorescence, but self-associates when entering the cells, resulting in a shift towards red fluorescence. The uptake of DiOC_2_ is promoted by Δψ (inside negative), thus cells with higher Δψ exhibit increased red fluorescence. *V*. *cholerae* Δ*pomAB* transformed with pAB, pAB-S26A or pAB-S26T, as well as *V*. *cholerae* Δ*nqr*, were streaked onto LB agar plates with appropriate antibiotics and cultivated over night at 37°C. 5 ml of LB medium with indicated salt composition and pH containing the appropriate antibiotics was inoculated with a single colony of each strain and incubated over night at 37°C / 180 rpm (orbit: 25 mm). 3 ml LB-Na^+^ or LB-K^+^ were inoculated with the overnight culture to an OD_600_ of 0.01 and incubated at 30°C / 180 rpm (orbit: 25 mm) for 4 h. Expression of His_6_-PomA and PomB-Strep (wild type or S26A and S26T variants) was started immediately by adding L-(+)-arabinose (final concentration: 10 mM). When LB medium without added salt was used, cultures were incubated at 30°C / 180 rpm for 5 h. The final OD_600_ of each culture was determined and the cultures were diluted in phosphate buffered saline (PBS; 10 mM Na-phosphate, 145 mM NaCl, pH 7.4) to a final OD_600_ of 0.1. Cells were washed in 1 ml PBS, sedimented by centrifugation (16.000 rpm, 15 min) and resuspended in 1 ml PBS. 5 μl DiOC_2_ (final concentration: 15 μM) was added to the cell suspensions from a 3 mM stock solution in DMSO. Cell-free buffer controls contained PBS with or without DiOC_2_. The cells were incubated at room temperature for 1 h. Subsequently, aliquots of 200 μl were transferred into a black, 96 well plate (4ti-0263, 4titude) to determine red (635 nm, band width 35 nm, gain: 49–53) fluorescence emission intensities using a TECAN Infinite F200 PRO plate reader. The excitation wavelength was 485 nm with a band width of 20 nm. After the measurements, the samples were transferred into a translucent, 96 well plate (4ti-0224, 4titude) and the OD_595_ was determined using the plate reader. Statistical analyses were performed using Origin (release 8.0). For each strain and condition, three biological and three technical replicates were performed. The fluorescence intensity of PBS without cells but with dye (background) was subtracted from the fluorescence intensities of the stained cell suspensions.

## Results

### Overproduction of PomA_4_B_2_ stator complexes and its variants in *V*. *cholerae* Δ*pomAB*

**Fig 2:**
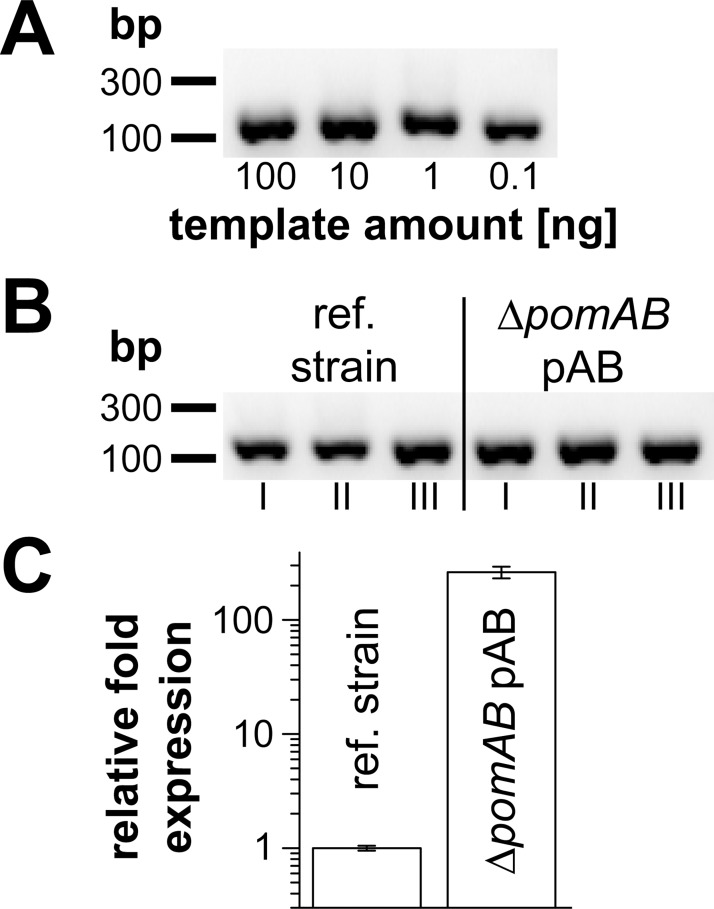
Transcription level of plasmid-encoded *versus* chromosomally encoded *pomB* in *V*. *cholerae* determined by qRT-PCR. A: analysis of amplification products obtained from qRT-PCR reactions with template (cDNA) amounts of 100 ng, 10 ng, 1 ng and 0.1 ng by agarose gel electrophoresis. The efficiency of the primer pair “pomB fwd” / “pomB rev” was 115% with R^2^ = 0.998, as calculated using the CFX Manager software (Bio-Rad). A 100 bp DNA ladder (GeneRuler, Thermo Scientific) served as molecular size marker. B: The correct size (134 bp) of the products from the qRT-PCR reactions (technical triplicates, I–III) was confirmed by agarose gel electrophoresis. C: Relative transcription level of *pomB* in the *V*. *cholerae* reference strain and in *V*. *cholerae* Δ*pomAB* pAB induced with 10 mM L-arabinose. For both strains, qRT-PCR reactions were performed in technical triplicates. Transcription levels of *pomB* were calculated using the CFX Manager software (Bio-Rad, version 2.1.1022.0523), and the transcription level of the chromosomally encoded *pomB* (reference strain) was set to 1.

**Fig 3:**
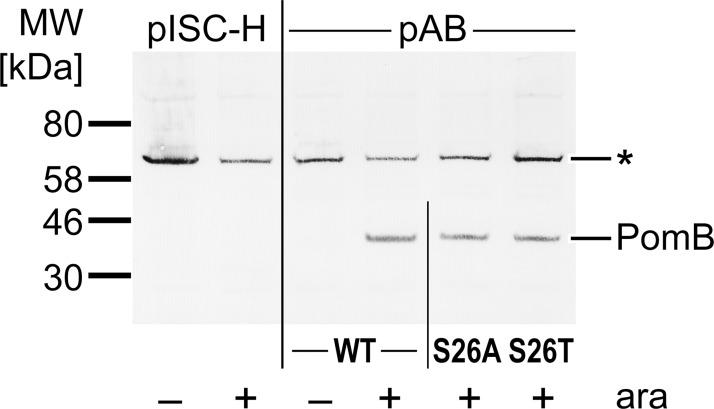
Expression levels of plasmid-encoded PomB variants in *V*. *cholerae* Δ*pomAB*. Wild type PomB-Strep, PomB-S26A-Strep and PomB-S26T-Strep were detected in extracts from *V*. *cholerae* Δ*pomAB* cells transformed with variants of plasmid pAB on a Western blot. PomB-Strep with an apparent molecular weight around 46 kDa was expressed in the presence (+) but not in the absence (‒) of 10 mM L-arabinose, and it was also absent in extracts from *V*. *cholerae* Δ*pomAB* transformed with the empty vector (pISC-H). Cells were grown for 4 h at 30°C, harvested and resuspended in SDS loading buffer to a final OD_600_ of 5.0. Per lane, an aliquot of 18 μg total cell protein was loaded. The bands (*) above the 58 kDa marker result from detection of an endogenous, biotinylated protein of *V*. *cholerae* by the Strep-Tactin-horseradish peroxidase conjugate [52].

**Fig 4:**
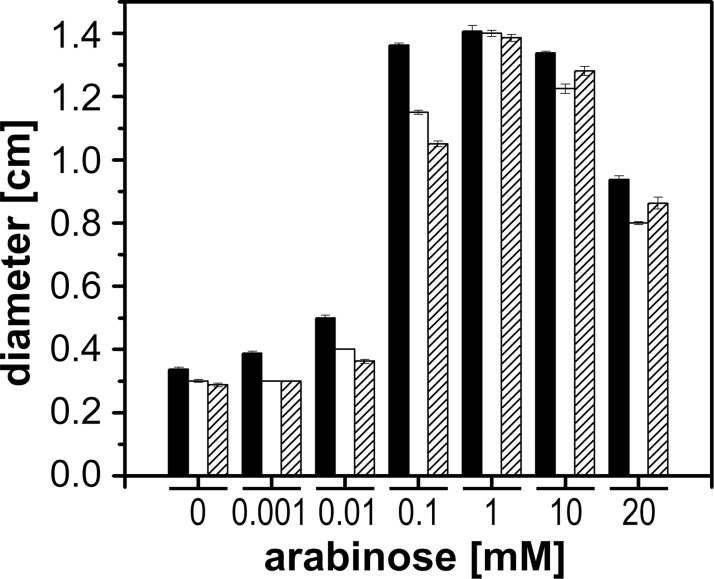
Influence of inducer concentration on motility of *V*. *cholerae* Δ*pomAB* overproducing His_6_-PomA together with PomB-Strep, PomB-S26A-Strep, or PomB-S26T-Strep. The average and standard deviations were calculated of eight experiments (the lowest and highest values from a total of ten experiments were omitted). Black bars: *V*. *cholerae* Δ*pomAB* pAB; open bars: *V*. *cholerae* Δ*pomAB* pAB-S26A; hatched bars: *V*. *cholerae* Δ*pomAB* pAB-S26T.

To estimate the level of overexpression of *pomB*, we performed qRT-PCR on *pomB*, using cDNA produced from total RNA of *V*. *cholerae* Δ*pomAB* transformed with plasmid pAB (Table 1). In the presence of 10 mM L-arabinose, the transcript levels were drastically increased (110-fold) in *V*. *cholerae* Δ*pomAB* transformed with pAB compared to the reference strain containing chromosomally encoded *pomAB* (Fig 2). This overexpression of *pomB* (together with *pomA*) from plasmid pAB is in accord with the previously observed accumulation of GFP-tagged stators at cell poles and at the inner membrane, using a plasmid derived from pAB in the same *V*. *cholerae* host, and with identical inducer concentration [52]. The strong expression of His_6_-PomA and PomB-Strep from plasmid pAB and its derivatives is also in accordance with our previous observation that production of PomB-Strep-D23N in the *V*. *cholerae* reference strain containing chromosomally encoded *pomB* resulted in drastically reduced motility compared to the reference strain transformed with the empty vector. PomB-D23N-Strep is an inactive variant of PomB-Strep which, together with His-tagged PomA, was inserted into the polar flagellum, replacing the endogenous, active PomA_4_PomB_2_ stators [29]. Likewise, introducing the D23E or D23N mutations did not alter the amount of overproduced PomB-Strep variant when compared to wild type PomB-Strep, as estimated from the intensity of PomB-Strep detected with Strep-Tactin-horseradish peroxidase conjugate on Western blots [29]. The present study showed that replacing S26 in PomB-Strep with A or T did not result in decreased expression when compared to wild type PomB-Strep (Fig 3). In accord with the Western blot analysis, similar high levels of mRNA of the mutated variants were assumed. Previously, GFP-tagged variants of PomB carrying the S26A and S26T mutations and wild type GFP-PomB were shown to exhibit similar expression levels and localization at the inner membrane [52]. The calculated molecular mass of PomB-Strep is 36.7 kDa, but the protein runs at an apparent molecular mass of almost 46 kDa on SDS-PAGE. In the absence of L-arabinose, *V*. *cholerae* did not produce PomB-Strep at detectable levels. The band at a molecular weight around 70 kDa represents a biotinylated protein from *V*. *cholerae* found in all strains in the absence or presence of inducer. Considering this strong overproduction of stator complexes in the presence of 10 mM L-arabinose, confirmed for PomB-Strep here on the level of mRNA, and previously on the level of protein [52], we conclude that overall motility of cells was not limited by the availability of pre-assembled stator complexes [58,59]. This was further confirmed in experiments designed to identify the concentration range of the inducer L-arabinose required for maximum motility on softagar plates (Fig 4). Motility was followed on LB-Na^+^ softagar plates at pH 8.0. When the L-arabinose concentration was raised from 0.01 to 0.1 mM, there was a steep increase in motility of *V*. *cholerae* Δ*pomAB* transformed with plasmids coding for His_6_-PomA together with wild type PomB-Strep, PomB-S26A-Strep or PomB-S26T-Strep (Fig 4). The largest motility rings were observed in the range from 1 to 10 mM L-arabinose. A further increase to 20 mM L-arabinose led to a decrease of motility in all strains. To study the effect of S26 mutations in PomB on motility of *V*. *cholerae*, tracking experiments were performed in the presence of 10 mM L-arabinose, as described in the following section.

### Tracking live *V*. *cholerae* cells by differential interference contrast microscopy

An important prerequisite for the tracking of swimming cells by light microscopy is a clear contrast between the individual cells and their surrounding space. In a previous study, we described the use of a fluorescent dye (MitoTracker, Invitrogen) to visualize *V*. *cholerae* cells by their fluorescence signal upon excitation with light of a distinct wavelength [29]. The advantage of this method is that it produces images of single cells which, at 200-fold magnification, appear as bright, point-shaped objects in front of a dark background. Such objects cannot only be safely detected by spot detection algorithms, they can also be tracked quite easily by tracking algorithms provided by commercially available image processing software like Volocity (Perkin Elmer, USA) or Imaris (Bitplane AG, Switzerland). However, there are disadvantages. One is that in the presence of the fluorescent dye, we observed a large fraction of immotile cells which produce significantly stronger fluorescent signals than motile cells do, and that these stronger signals tend to overlay the weaker signals of swimming cells, especially those of fast swimming cells. This particularly becomes a problem when a small fraction of fast swimming cells is to be analyzed in the presence of a considerable number of non-swimming cells. Another disadvantage is that the fluorescence signal from MitoTracker stained cells bleaches out quite rapidly, preventing the recording of video sequences over longer periods of time. The method described here allows determination of velocities of a large number of cells in the absence of fluorescent dyes, i.e. under physiological conditions. The majority of cells (>90%) is motile under all conditions tested here. Tracking of cells is not impaired by bleaching of the fluorophore, and there is no background fluorescence caused by immotile cells which could interfere with the automated tracking of motile cells.

### Overproduction of PomA and PomB promotes fast swimming of *V*. *cholerae*

**Fig 5:**
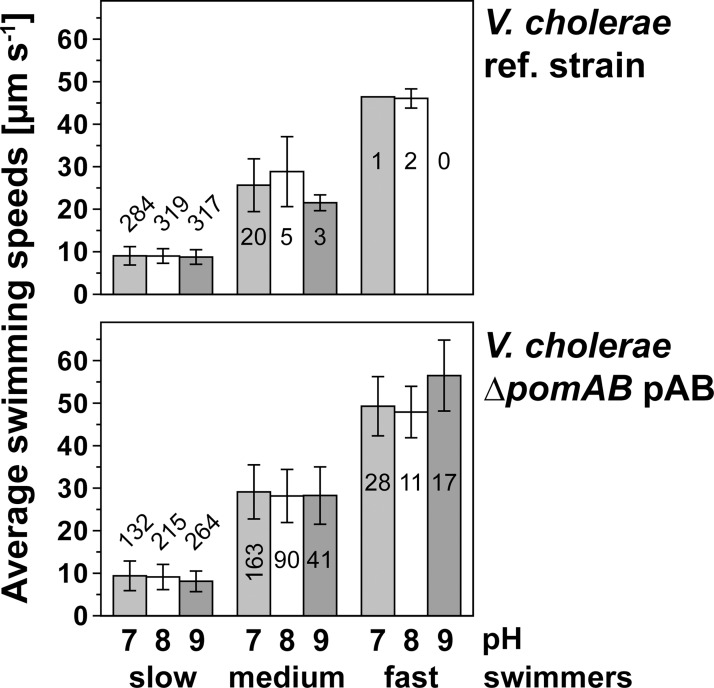
Distribution of swimming speeds of the *V*. *cholerae* reference strain and of *V*. *cholerae* Δ*pomAB* expressing *pomA* and *pomB* in trans. Tracks were recorded in LB-Na^+^ adjusted with Tris to pH 7.0 (light grey bars), pH 8.0 (white bars), or pH 9.0 (dark grey bars). Tracking results of individual cells were assigned to three main classes of velocities (“slow”, “medium” and “fast”). The numbers in the bars indicate the number of tracks used to calculate the mean values and standard deviations. Minimum, median and maximum values are given in the supporting information (S1 Table).

**Fig 6:**
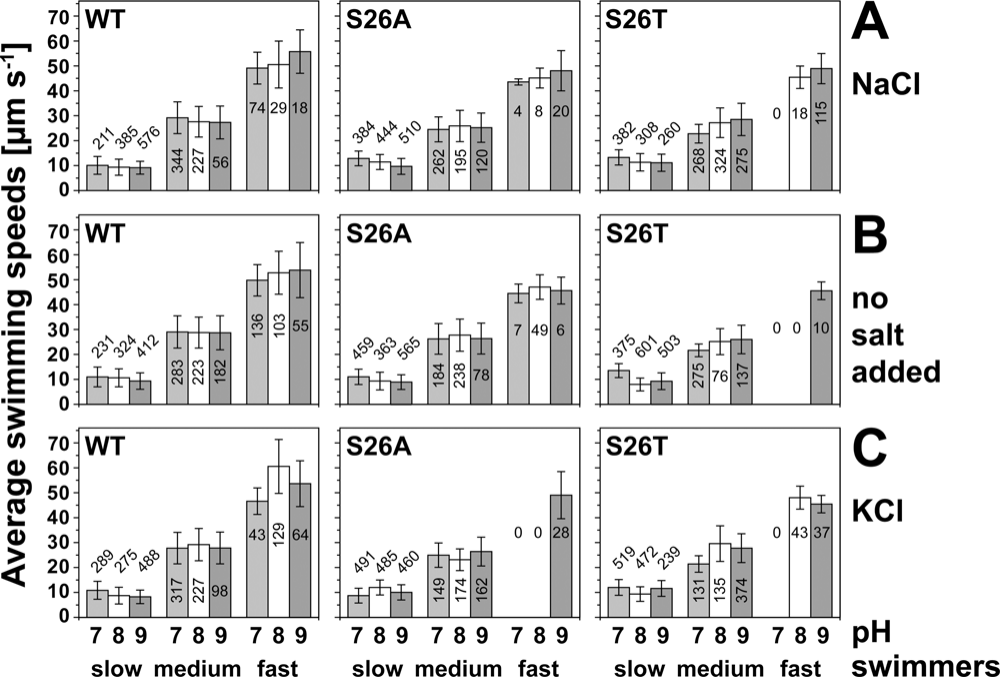
Influence of pH and salt condition on the swimming speeds of *V*. *cholerae* Δ*pomAB* cells overproducing His_6_-PomA together with PomB-Strep, PomB-S26A-Strep, or PomB-S26T-Strep. Tracks were recorded in different LB media adjusted with Tris to pH 7.0 (light grey bars), pH 8.0 (white bars), or pH 9.0 (dark grey bars). A: LB-Na^+^; B: no salt added, with residual concentrations of 11 mM Na^+^ and 12 mM K^+^; C: LB-K^+^. Tracking results of individual cells were assigned to three main classes of velocities (“slow”, “medium” and “fast”). The numbers in the bars represent the number of tracks recorded under the specified condition. The mean values and standard deviations of velocities are presented. Minimum, median and maximum values are given in the supporting information (S2–S4 Tables).

There is a continuous replacement of stators in the flagellum using pre-assembled PomA_4_B_2_ complexes [37,59]. With a vector system for low expression of PomA and PomB, a limitation in stator complexes could decrease motility compared to the reference strain with endogenous *pomA* and *pomB* genes. On the other hand, a vector allowing for high expression could result in an overload of pre-assembled stator complexes, possibly interfering with the insertion of the stators, and impairing flagellar function. Using a low-expression vector for the production of stator complexes, Berry and coworkers [58] observed a distribution of rotation speeds using beads attached to a single, truncated flagellar filament of an *E*. *coli* cell fixed to a glass surface. The rotational speeds were presented in histograms, and the authors assumed that the distinct frequencies resulted from different numbers of stator units present in the flagellar motor of the cell. In that study which was performed with a chimeric, Na^+^-dependent stator, the slowest major peak in each histogram was assigned to the frequence of a flagellar motor containing a single stator unit [58]. In our previous studies [29,52], we found no evidence for an inhibition of motility of *V*. *cholerae* Δ*pomAB* overexpressing its endogenous, Na^+^-dependent stator complexes at an inducer concentration of 10 mM L-arabinose, a finding which is further substantiated in motility experiments on soft agar plates (Fig 4). The fastest swimmers among these cells were expected to operate flagellar motors with maximum occupancy of stator complexes. We tested if overproduction of the PomA_4_PomB_2_ stator in *V*. *cholerae* Δ*pomAB* had an effect on the swimming speed of individual cells when compared to the reference strain. Irrespective of the strain and the pH (7.0, 8.0, or 9.0) of the medium studied, *V*. *cholerae* cells exhibited large variations in swimming speeds. For better comparison of the results, velocity classes from 4 to <18 μm s^-1^ (slow swimmers), 18 to <41 μm s^-1^ (medium swimmers), or 41 μm s^-1^ and faster (fast swimmers) were defined (Fig 5). In Fig 5, the numbers in the bars represent the number of tracks analyzed to obtain the mean velocity and standard deviation of the given class. We also determined the median of velocities of a given class, yielding a similar result with respect to interpretation of the data (S1 Table). In LB-Na^+^ medium, the majority of the analyzed cells of the *V*. *cholerae* reference strain (wild type) exhibited slow swimming speeds between 4 μm s^-1^ and <18 μm s^-1^ at all proton concentrations of the medium tested (Fig 5). In LB-Na^+^, the greatest speed determined for the *V*. *cholerae* reference strain (48 μm s^-1^) was observed in medium buffered to pH 8.0 (S1 Table). However, only a small subset of cells exhibited high velocities at pH 7.0 and 8.0, while the majority fell into the medium and, first of all, into the slow swimmer classes. When the pH was raised to 9.0, we did not observe any cells faster than 23 μm s^-1^ (S1 Table). Interestingly, a different distribution of swimming speeds was observed with the *V*. *cholerae* Δ*pomAB* strain expressing plasmid-encoded wild type His_6_-PomA and PomB-Strep, containing increased amounts of PomA_4_PomB_2_ stator complexes (Fig 5). At all pH values tested (7.0, 8.0 and 9.0), the *V*. *cholerae* Δ*pomAB* strain, transformed with plasmid pAB encoding for His-tagged *pomA* and Strep-tagged *pomB*, exhibited higher motilities than the reference strain. This is most obvious when comparing either the distribution of cells over the three main classes of swimming speeds, or their top speeds. Particularly, the medium (18 μm s^-1^ to <41 μm s^-1^) as well as the fast (41 μm s^-1^ or faster) speed classes were much more populated by the complemented *pomAB* deletion strain than by the reference strain (Fig 5). Whereas top swimming speeds of up to 61 μm s^-1^ (LB-Na^+^ pH 8.0), 73 μm s^-1^ (LB-Na^+^ pH 9.0) or even 74 μm s^-1^ (LB-Na^+^ pH 7.0) were observed with complemented Δ*pomAB* cells, the fastest cells of the reference strain were slower (48 μm s^-1^ at pH 8.0, 40 μm s^-1^ at pH 7.0), especially at pH 9.0 (23 μm s^-1^) (S1 Table). It is noteworthy that the highest speed (94 μm s^-1^) of *V*. *cholerae* Δ*pomAB* transformed with plasmid pAB was observed in LB-K^+^ (pH 8.0) (Fig 6, and S2–S4 Tables), but not in LB-Na^+^. In summary, the endogenous levels of PomA and PomB in the *V*. *cholerae* reference strain were insufficient to promote maximum velocity, at least under the chosen conditions (LB medium with Tris buffer). Therefore, overexpression from plasmid pAB was a prerequisite to study the effect of mutations within the Na^+^ channels on fast swimming, as described in the following section.

### S26 in PomB is critical for fast swimming of *V*. *cholerae* cells

We previously showed that motility of *V*. *cholerae* cells critically depends on osmolality and pH [52]. The analysis of a large set of cell tracks, followed by assignment to distinct classes of velocities, revealed an unexpected phenotype of cells carrying mutations at position S26 of PomB from *V*. *cholerae*. We recorded at least 631 tracks per strain and condition to compare the effect of salt and pH on the swimming speeds of *V*. *cholerae* Δ*pomAB* cells expressing wild type PomB-Strep, or its S26T or S26A variants. With cells expressing wild type stator complexes, the highest velocities (top speed as well as average and median speed of the fast swimmer classes), and the second largest number of fast swimmers, were observed at pH 8.0 in the presence of 11 mM Na^+^ and 183 mM K^+^ (Fig 6, and S2–S4 Tables). High Na^+^ (182 mM, with 12 mM K^+^) was inhibitory, since the lowest numbers (29 at pH 8.0, 18 at pH 9.0) of fast swimmers were observed under this condition of salt. Without any added salt, the numbers of fast swimmers at pH 8.0 or 9.0 lay between the numbers observed with LB-Na^+^ and LB-K^+^ (Fig 6, and S2–S4 Tables). This is in accord with our previous findings that the increase in osmolality (due to added chloride salts) rather than the increase in Na^+^ concentration promoted motility of *V*. *cholerae* cells overexpressing wild type stator complexes [52]. Chloride, a chaotropic anion, might distort the local water structure within the Na^+^ access channel of the PomA_4_PomB_2_ stator, partially disrupting the hydration shell of Na^+^, and facilitating its passage through the channel. We next focused on the results obtained with *V*. *cholerae* cells at pH 7.0 since this was the pH chosen for the *V*. *alginolyticus* study addressing the effect of the PomB-S27T mutation [53]. Fast swimmers were detected among the *V*. *cholerae* Δ*pomAB* cells expressing wild type PomB-Strep under all salt conditions tested (182 mM Na^+^/12 mM K^+^, 11 mM Na^+^/12 mM K^+^, or 11 mM Na^+^/183 mM K^+^). With cells expressing the PomB-S26T-Strep variant, the ability for fast swimming (41 μm s^-1^ or faster) was lost under all salt conditions at pH 7.0. Raising the pH to 9.0 rescued cells producing PomB-S26T-Strep, enabling them for fast swimming under all salt concentrations studied (Fig 6), with distributions of velocities similar to wild type cells. However, at pH 8.0, the presence of salt became critical for the stator containing the PomB-S26T-Strep variant. Fast swimming of cells required the presence of either 182 mM Na^+^/12 mM K^+^ or 11 mM Na^+^/183 mM K^+^. At 11 mM Na^+^/12 mM K^+^ (LB without added salt), no fast swimmers were observed at all (Fig 6, and S2–S4 Tables). When S26 was replaced by alanine, fast swimming cells (comparable to the wild type) were observed at pH 7.0, 8.0 and 9.0 at low salt (11 mM Na^+^/12 mM K^+^) or high Na^+^ (182 mM Na^+^/12 mM K^+^), but not at high K^+^ (11 mM Na^+^/183 mM K^+^) with pH 7.0 or 8.0. Only at pH 9.0, cells possessing the PomB-S26A-Strep variant exhibited fast swimming despite the presence of 183 mM K^+^ (Fig 6).

### Formation of transmembrane voltage in *V*. *cholerae* Δ*pomAB* expressing PomB and its S26A or S26T variants

**Fig 7:**
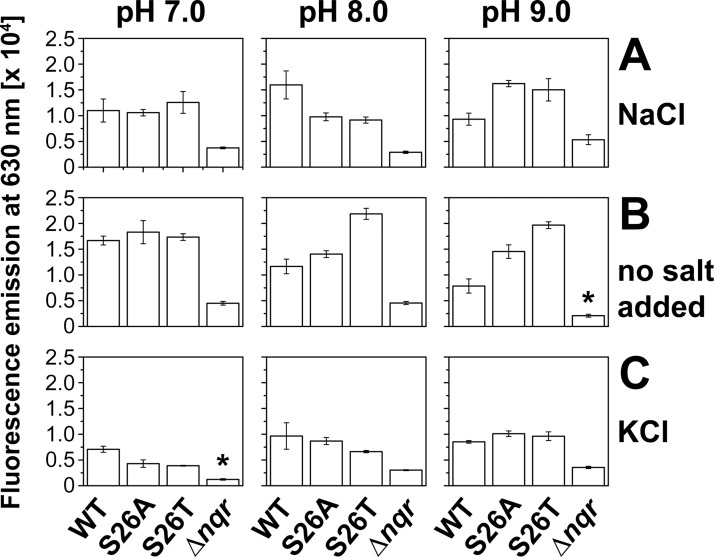
Influence of pH and salt condition on the membrane potential generated by *V*. *cholerae* Δ*pomAB* cells overproducing His_6_-PomA together with PomB-Strep, PomB-S26A-Strep, or PomB-S26T-Strep. The fluorescence emission of the fluorophore DiOC_2_ at 630 nm was determined to estimate the formation of membrane potential, ΔΨ (inside negative). Average values and standard deviations of three biological replicates, each from a different culture, are presented. *V*. *cholerae* Δ*nqr* lacks the Na^+^-translocating NADH:quinone oxidoreductase and is impaired in the formation of ΔΨ. The asterisks indicate conditions which allow only poor growth of *V*. *cholerae* Δ*nqr*, impairing determination of ΔΨ. A: LB-Na^+^; B: no salt added, with residual concentrations of 11 mM Na^+^ and 12 mM K^+^; C: LB-K^+^.

Overexpression resulted in the accumulation of pre-assembled stators in the inner membrane of *V*. *cholerae* [52]. Unspecific ion flux ‒ especially when introducing mutations within the channel region of the stators ‒ should be considered as this might dissipate the sodium-motive force across the inner membrane. This in turn could affect flagellar rotation. The MotA_4_MotB_2_ stators of the proton-dependent flagella of *E*. *coli* and *Salmonella*, as well as the PomA_4_PomB_2_ stators of the sodium-dependent flagella of *V*. *alginolyticus*, assume a “plugged” confirmation in their pre-assembled state and therefore do not promote ion flux prior to insertion into the flagellar motor [60–65]. To exclude that the overexpressed stators containing PomB variants led to a decrease in the sodium-motive force, the transmembrane voltage established by the different *V*. *cholerae* strains was determined. As a control, *V*. *cholerae* Δ*nqr* lacking the Na^+^-translocating NADH:quinone oxidoreductase (Na^+^-NQR) was included in the analysis. The Na^+^-NQR is an electrogenic NADH dehydrogenase in the respiratory chain of *V*. *cholerae* and represents the main generator of the sodium-motive force [66]. Cells were grown in LB medium at the indicated conditions, and expression of wild type PomB-Strep, PomB-S26A-Strep or PomB-S26T-Strep in the *V*. *cholerae* Δ*pomAB* host was induced by adding 10 mM L-arabinose. The *V*. *cholerae* Δ*nqr* strain devoid of the Na^+^-NQR was included to demonstrate that a decrease in membrane potential was readily detected with the BacLight assay. This deletion strain lacks its respiratory Na^+^ pump which contributes to the formation of a transmembrane voltage and a chemical Na^+^ gradient in *V*. *cholerae*. Yet, *V*. *cholerae* Δ*nqr* is viable and motile, and contains all flagellar genes [67]. With all PomB variants studied, and under all conditions of salt and pH investigated, the transmembrane voltage established by *V*. *cholerae* strains overproducing PomA_4_B_2_ stator complexes was always higher than the transmembrane voltage established by the *nqr* deletion strain (Fig 7). We conclude that the presence of pre-assembled stator complexes in the inner membrane [52] did not diminish the sodium-motive force by passive influx of Na^+^ into the cell. The transmembrane voltage established by *V*. *cholerae* Δ*pomAB* expressing the PomB-S26A-Strep or PomB-S26T-Strep did not significantly differ from ΔΨ established by cells expressing wild type PomB-Strep. It is noteworthy that at pH 7.0, the S26T variant did not confer any drastic change in ΔΨ under every salt condition tested when compared to wild type PomB-Strep, but was clearly impaired in motility (Fig 6). High K^+^ at pH 7.0 or 8.0 was inhibitory for fast swimming in the PomB-S26A-Strep variant (Fig 6), but cells expressing PomB-S26A-Strep were not compromised in ΔΨ formation under these conditions. In summary, we demonstrated that introducing mutations did not affect the expression level of PomB-Strep, and did not diminish the transmembrane voltage required to drive flagellar rotation. The effects of mutations in PomB on motility described here therefore resulted from altered cation transport properties of the PomA_4_B_2_ complex as part of the flagellar motor.

## Discussion

**Fig 8:**
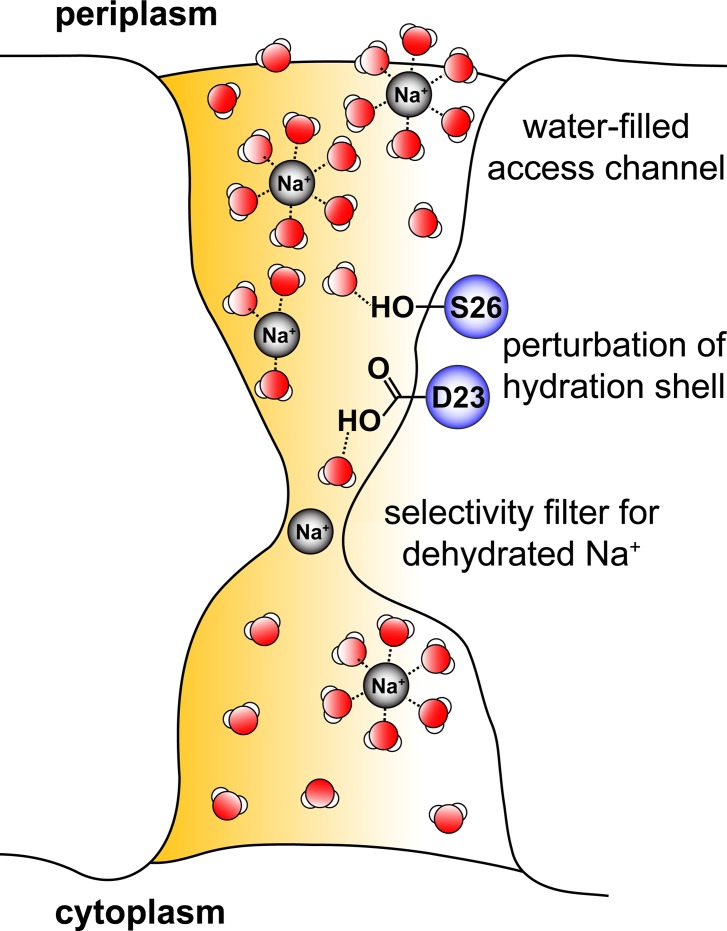
Na^+^ flux through the flagellar PomA_4_PomB_2_ complex by a channel-like mechanism. Na^+^ enters the PomA_4_PomB_2_ stator complex in its hydrated state from the periplasm via a water-filled access channel. The conserved amino acid residues S26 and D23 within the membrane-bound region of PomB interact with water molecules surrounding the central Na^+^, hereby perturbing its hydration shell. The sodium ion passes the narrow constriction site, or selectivity filter, in its dehydrated state. After passage through the filter, Na^+^ is hydrated and released towards the cytoplasmic side of the stator. The downhill flux of Na^+^ from the periplasm to the cytoplasm is driven by the electrochemical Na^+^ gradient across the inner membrane.

The PomA_4_PomB_2_ complex of the polar flagellum of *V*. *cholerae* provides at least one pathway for the transport of Na^+^ from the periplasm to the cytoplasm along the electrochemical Na^+^ gradient. This so-called stator complex is an essential part of the motor, since the flux of Na^+^ through PomA_4_PomB_2_ is a prerequisite for rotation of flagellum [6,52,59,68] (Fig 1). The terms “Na^+^ transport” or “Na^+^ flux” suggest different mechanisms of stator function. Does PomA_4_PomB_2_ represent a Na^+^ transporter (or pump) with a distinct binding site for Na^+^, exemplified by the F_o_ part of the Na^+^-translocating F_1_F_o_ ATP synthase [69]? Or does it bear similarity to a channel designed for the fast flux of cations, an example being the K^+^ channel [70]? Assuming that the stator complex possesses a critical carboxylate required as a ligand for Na^+^ in a binding site, as found in the F_o_ part of the ATP synthase, one would expect a competition between Na^+^ and H^+^ for binding to this carboxylate. Increasing the pH would promote flagellar rotation at a fixed Na^+^ concentration. On the other hand, increasing the Na^+^ concentration at a given pH should prevent protonation of the critical carboxylate. However, with the Na^+^-dependent flagellum from *V*. *cholerae*, rotation was enhanced by a raise in external proton concentration, and Na^+^ did not prevent the protonation of membrane-embedded carboxylates, indicated by the covalent modification of the carboxylic groups with carbodiimide [52]. These and other findings pointed against a direct binding of Na^+^ to the critical, membrane-embedded D23 in PomB. A direct binding of Na^+^ was previously proposed for the corresponding D24 in the related PomB from *V*. *alginolyticus* [71]. Yet doubtlessly, this strictly conserved aspartate in PomB plays an important role during Na^+^ transport. Replacing D23 in *V*. *cholerae* PomB with asparagine, or the corresponding D24 in *V*. *alginolyticus* PomB with asparagine or cysteine, completely blocks flagellar rotation [29,30,40,52]. In the PomA wild type background, the presence of a carboxyl group (provided by Asp or Glu) at this position in PomB is essential for motor function [29,52,72]. By studying the motility behavior of *V*. *cholerae* expressing different variants of PomB under different conditions of salt and pH in softagar plates, we proposed that D23 and S26 in the membrane-embedded stretch of PomB contribute to the formation of a hydrogen bond network which is essential for passage of Na^+^ through the stator [52]. Our results suggested that the protonation of D23, or its interaction with water or hydronium ions, is still possible despite the presence of Na^+^ [52]. The specificity of the PomA_4_PomB_2_ stator for Na^+^ implies that at a certain step in the transport process, the cation must become at least partially dehydrated [70]. Otherwise, the stator complex could not discriminate Na^+^ from other cations, like K^+^. Once the water molecules are (partially) removed, the PomA_4_PomB_2_ stator may offer distinct binding site(s) which satisfy the need of the sodium ion with respect to its coordination geometry (if operating as a pump). Alternatively, the stator may provide a selectivity filter, which only allows passage of the (at least partially dehydrated) sodium ion (if operating as a channel) [70,73]. Berry and coworkers [58] studied the performance of flagellum rotation in *E*. *coli* using a chimeric stator providing specificity towards Na^+^ (and not H^+^) as coupling ion for the flagellar motor. *E*. *coli* cells were fixed to a glass surface, and motor rotation was observed via back-focal-plane interferometry of beads attached to a single, truncated flagellar filament. Using a low-expression vector for stator production, authors of that study observed distributions of rotational speeds which were presented in histograms. It was assumed that the distinct rotational frequencies resulted from different numbers of stator complexes integrated in the flagellar motor of the particular cell studied, and the slowest major peak in each histogram was assigned to the rotational frequency of a flagellar motor equipped with one, single stator unit [58]. In our investigations, we chose a high-expression system for the Na^+^-dependent stator from *V*. *cholerae* studied in complex with its endogenous flagellar motor in *V*. *cholerae*, focusing on the fast swimmers which operate flagella with maximum occupancy of stator complexes. These fast swimmers were only observed with overproduced stator complexes, and enabled us to study the influence of pH and salt composition of the medium on the performance of stators containing wild type PomB (fused to a Strep tag), or variants carrying mutations in PomB-Strep. S26 within the single transmembrane helix of PomB was critical for fast rotation of the flagellum under a broad range of physiological H^+^ and salt concentrations. Removal of the hydroxyl group at this position (S26A), or even maintaining the OH-group, but at a slightly different position (S26T), strongly impaired fast swimming of *V*. *cholerae* cells, but in a pH- and salt-dependent manner. Homma and coworkers (2011) studied the effect of the corresponding S27T mutation in PomB of *V*. *alginolyticus* and observed some contribution of S27T to rotor function in the presence of 200 mM Na^+^ [53]. With *V*. *cholerae*, cells expressing the PomB-S26T-Strep variant were clearly slowed down compared to cells with wild type PomB-Strep at pH 7.0, but regained the ability for fast swimming at pH 9.0. This strong effect is difficult to reconcile with different protonation states of the OH-groups in serine or threonine residues at a given pH in the range from 7.0 to 9.0, since the pK_a_ of the hydroxyl group in both amino acids is too high (>13) to expect deprotonated states. Instead, the observed effects of salt and H^+^ concentrations on stator function could be due to a rearrangement of water molecules or hydrogen bonds within the water-filled access channel of the stator complex (Fig 8). A helical wheel prediction of PomB suggests that S26 resides on the same side as the critical D23 and faces the inside of a transmembrane channel built by PomA_4_PomB_2_ [32,52]. D23 is positioned below S26 when viewed from the periplasmic side of the inner membrane (Fig 8). It is proposed that S26, together with D23, is important for fast transport through the stator channel by perturbing the hydration shell of Na^+^, preparing it for passage through a selectivity filter located below D23. This constriction in the passageway for the Na^+^ will not depend on the presence of charged amino acid side chains as long as the critical diameter is maintained which is selective for dehydrated Na^+^ (Fig 8). This model would be consistent with the finding that studies with chimeric stators made up of domains from subunits derived from H^+^- or Na^+^-dependent stators so far failed to identify critical amino acids conferring cation selectivity [24]. It should be noted that D23 could also be part of the selectivity filter which might accommodate a partially hydrated Na^+^, as proposed for the bacterial Na^+^ channel [73]. This scenario cannot unequivocally be excluded by our findings. In summary, we demonstrated that S26 is crucial to promote fast rotation of the flagellum by facilitating sodium ion transport across the cell membrane. Our results indicate that S26 does not directly interact with Na^+^ but rather affects its hydration shell. S26 of PomB is important for Na^+^ flux through PomA_4_B_2_ under very different conditions of salinity and pH, and guarantees motility of *V*. *cholerae* in low- and high-salinity environments [74] inhabited by this versatile pathogen.

## Supporting Information

S1 Table Statistical analyses of tracks from *V*. *cholerae* reference strain and *V*. *cholerae* Δ*pomAB* transformed with pAB coding for His_6_-*pomA* and *pomB*-Strep. Velocities were determined in LB medium with 171 mM Na^+^ added. At pH 7.0, 8.0 and 9.0, the total numbers of tracks recorded with the *V*. *cholerae* reference strain were 305, 326 and 320, and with *V*. *cholerae* Δ*pomAB* expressing His_6_-PomA and wild type PomB-Strep 323, 316 and 322, respectively. SD: Standard deviation.
(PDF)

S2 Table Statistical analyses of tracks from *V*. *cholerae* Δ*pomAB* cells overproducing His_6_-PomA together with PomB-Strep, PomB-S26A-Strep or PomB-S26T-Strep in LB medium with 171 mM Na^+^ added. At pH 7.0, 8.0 and 9.0, the total numbers of tracks recorded with *V*. *cholerae* Δ*pomAB* expressing His_6_-PomA and wild type PomB-Strep were 629, 641 and 650, respectively. For His_6_-PomA together with PomB-S26A-Strep, the numbers of tracks were 650, 647 and 650, respectively, and 650, 650 and 650 for His_6_-PomA together with PomB-S26T-Strep. SD: Standard deviation.
(PDF)

S3 Table Statistical analyses of tracks from *V*. *cholerae* Δ*pomAB* cells overproducing His_6_-PomA together with PomB-Strep, PomB-S26A-Strep or PomB-S26T-Strep in LB medium without added salt. At pH 7.0, 8.0 and 9.0, the total numbers of tracks recorded with *V*. *cholerae* Δ*pomAB* expressing His_6_-PomA and wild type PomB-Strep were 650, 650 and 649, respectively. For His_6_-PomA together with PomB-S26A-Strep, the numbers of tracks were 650, 650 and 649, respectively, and 650, 677 and 650 for His_6_-PomA together with PomB-S26T-Strep. SD: Standard deviation.
(PDF)

S4 Table Statistical analyses of tracks from *V*. *cholerae* Δ*pomAB* cells overproducing His_6_-PomA together with PomB-Strep, PomB-S26A-Strep or PomB-S26T-Strep in LB medium with 171 mM K^+^ added. At pH 7.0, 8.0 and 9.0, the total numbers of tracks recorded with *V*. *cholerae* Δ*pomAB* expressing His_6_-PomA and wild type PomB-Strep were 649, 631 and 650, respectively. For His_6_-PomA together with PomB-S26A-Strep, the numbers of tracks were 640, 659 and 650, respectively, and 650, 650 and 650 for His_6_-PomA together with PomB-S26T-Strep. SD: Standard deviation.
(PDF)
