# Supplementary material for: Serine 26 in the PomB Subunit of the Flagellar Motor Is Essential for Hypermotility of Vibrio cholerae
Source: PLoS One. 2015 Apr 15;10(4):e0123518. doi: 10.1371/journal.pone.0123518 (PMC4398553; doi:10.1371/journal.pone.0123518)
Supplement: S1 Table — Velocities were determined in LB medium with 171 mM Na+ added. At pH 7.0, 8.0 and 9.0, the total numbers of tracks recorded with the V. cholerae reference strain were 305, 326 and 320, and with V. cholerae ΔpomAB expressing His6-PomA and wild type PomB-Strep 323, 316 and 322, respectively. SD: Standard deviation. (PDF) [file pone.0123518.s001.pdf]

Medium	LB with 171 mM Na^+ added (LB-Na^+)								
**Strain**	***V. cholerae* reference strain**								
**Class**	**slow**			**medium**			**fast**		
**Speed range**	**4 – <18 µm s^{-1}**			**18 – <41 µm s^{-1}**			**41 µm s^{-1} or faster**		
**pH**	**7.0**	**8.0**	**9.0**	**7.0**	**8.0**	**9.0**	**7.0**	**8.0**	**9.0**
Number of tracks	284	319	317	20	5	3	1	2	0
Average [µm s^{-1}]	9.04	9.00	8.75	25.64	28.84	21.53	45.44	46.06	0.00
SD [µm s^{-1}]	2.15	1.72	1.72	6.20	8.24	1.86	0.00	2.26	0.00
Minimum [µm s^{-1}]	6.23	6.43	6.56	18.30	19.05	19.37	45.44	44.46	0.00
Median [µm s^{-1}]	8.41	8.59	8.29	23.11	33.02	21.37	45.44	46.06	0.00
Maximum [µm s^{-1}]	17.81	17.69	17.77	40.09	37.29	23.46	45.44	47.66	0.00
**Strain**	***V. cholerae* Δ*pomAB* pAB**								
**Class**	**slow**			**medium**			**fast**		
**Speed range**	**4 – <18 µm s^{-1}**			**18 – <41 µm s^{-1}**			**41 µm s^{-1} or faster**		
**pH**	**7.0**	**8.0**	**9.0**	**7.0**	**8.0**	**9.0**	**7.0**	**8.0**	**9.0**
Number of tracks	132	215	264	163	90	41	28	11	17
Average [µm s^{-1}]	9.41	9.12	8.11	29.14	28.17	28.29	49.27	47.92	56.49
SD [µm s^{-1}]	3.49	2.95	2.41	6.37	6.25	6.74	6.96	6.04	8.33
Minimum [µm s^{-1}]	5.36	5.45	4.86	18.16	18.29	18.07	42.16	41.68	42.49
Median [µm s^{-1}]	7.93	7.98	7.17	28.76	26.91	27.98	47.77	47.52	56.11
Maximum [µm s^{-1}]	17.74	17.67	17.47	40.83	40.32	40.02	74.32	61.06	73.15
