# Supplementary material for: Serine 26 in the PomB Subunit of the Flagellar Motor Is Essential for Hypermotility of Vibrio cholerae
Source: PLoS One. 2015 Apr 15;10(4):e0123518. doi: 10.1371/journal.pone.0123518 (PMC4398553; doi:10.1371/journal.pone.0123518)
Supplement: S2 Table — At pH 7.0, 8.0 and 9.0, the total numbers of tracks recorded with V. cholerae ΔpomAB expressing His6-PomA and wild type PomB-Strep were 629, 641 and 650, respectively. For His6-PomA together with PomB-S26A-Strep, the numbers of tracks were 650, 647 and 650, respectively, and 650, 650 and 650 for His6-PomA together with PomB-S26T-Strep. SD: Standard deviation. (PDF) [file pone.0123518.s002.pdf]

**Medium**	**LB with 171 mM Na^+ added (LB-Na^+)**								
**Strain**	***V. cholerae* Δ*pomAB* pAB**								
**Class**	**slow**			**medium**			**fast**		
**Speed range**	**4 – <18 μm s^{-1}**			**18 – <41 μm s^{-1}**			**41 μm s^{-1} or faster**		
**pH**	**7.0**	**8.0**	**9.0**	**7.0**	**8.0**	**9.0**	**7.0**	**8.0**	**9.0**
Number of tracks	211	385	576	344	227	56	74	29	18
Average [μm s^{-1}]	10.09	9.34	9.15	29.20	27.59	27.32	49.10	50.54	55.73
SD [μm s^{-1}]	3.58	3.20	2.56	6.37	6.15	6.59	6.40	9.40	8.71
Minimum [μm s^{-1}]	5.36	5.36	4.86	18.14	18.00	18.07	41.27	41.45	42.49
Median [μm s^{-1}]	8.73	8.16	8.66	28.54	26.54	25.6	47.07	47.52	55.75
Maximum [μm s^{-1}]	17.74	17.91	17.91	40.96	40.97	40.02	74.31	82.27	73.16
**Strain**	***V. cholerae* Δ*pomAB* pAB-S26A**								
**Class**	**slow**			**medium**			**fast**		
**Speed range**	**4 – <18 μm s^{-1}**			**18 – <41 μm s^{-1}**			**41 μm s^{-1} or faster**		
**pH**	**7.0**	**8.0**	**9.0**	**7.0**	**8.0**	**9.0**	**7.0**	**8.0**	**9.0**
Number of tracks	384	444	510	262	195	120	4	8	20
Average [μm s^{-1}]	12.87	11.44	9.68	24.52	25.91	25.23	43.56	45.12	48.04
SD [μm s^{-1}]	2.94	3.02	3.21	5.00	6.29	5.86	1.24	4.00	8.08
Minimum [μm s^{-1}]	6.96	5.22	4.94	18.01	18.02	18.06	42.15	42.18	41.23
Median [μm s^{-1}]	12.75	11.02	8.71	23.64	24.84	23.11	43.45	43.31	45.29
Maximum [μm s^{-1}]	17.99	17.97	17.82	40.49	40.54	40.17	45.16	52.09	65.45
**Strain**	***V. cholerae* Δ*pomAB* pAB-S26T**								
**Class**	**slow**			**medium**			**fast**		
**Speed range**	**4 – <18 μm s^{-1}**			**18 – <41 μm s^{-1}**			**41 μm s^{-1} or faster**		
**pH**	**7.0**	**8.0**	**9.0**	**7.0**	**8.0**	**9.0**	**7.0**	**8.0**	**9.0**
Number of tracks	382	308	260	268	324	275	0	18	115
Average [μm s^{-1}]	13.29	11.33	11.16	22.80	27.19	28.52	0.00	45.45	48.92
SD [μm s^{-1}]	3.07	3.5	3.43	3.74	5.96	6.47	0.00	4.50	6.06
Minimum [μm s^{-1}]	6.24	5.58	5.18	18.03	18.03	18.03	0.00	41.21	41.11
Median [μm s^{-1}]	13.73	10.88	10.77	22.03	26.68	28.37	0.00	44.10	47.78
Maximum [μm s^{-1}]	17.99	17.99	17.93	37.42	40.97	40.94	0.00	60.78	75.81
