# Supplementary material for: Serine 26 in the PomB Subunit of the Flagellar Motor Is Essential for Hypermotility of Vibrio cholerae
Source: PLoS One. 2015 Apr 15;10(4):e0123518. doi: 10.1371/journal.pone.0123518 (PMC4398553; doi:10.1371/journal.pone.0123518)
Supplement: S3 Table — At pH 7.0, 8.0 and 9.0, the total numbers of tracks recorded with V. cholerae ΔpomAB expressing His6-PomA and wild type PomB-Strep were 650, 650 and 649, respectively. For His6-PomA together with PomB-S26A-Strep, the numbers of tracks were 650, 650 and 649, respectively, and 650, 677 and 650 for His6-PomA together with PomB-S26T-Strep. SD: Standard deviation. (PDF) [file pone.0123518.s003.pdf]

Medium	LB without added salt								
**Strain**	***V. cholerae* Δ*pomAB* pAB**								
**Class**	**slow**			**medium**			**fast**		
**Speed range**	**4 – <18 µm s^{-1}**			**18 – <41 µm s^{-1}**			**41 µm s^{-1} or faster**		
**pH**	**7.0**	**8.0**	**9.0**	**7.0**	**8.0**	**9.0**	**7.0**	**8.0**	**9.0**
Number of tracks	231	324	412	283	223	182	136	103	55
Average [µm s^{-1}]	10.97	10.61	9.33	29.01	28.78	28.69	49.75	52.78	53.83
SD [µm s^{-1}]	3.98	3.62	3.28	6.46	6.22	6.82	6.28	8.61	11.04
Minimum [µm s^{-1}]	4.13	4.72	4.77	18.13	18.03	18.24	41.12	41.08	41.04
Median [µm s^{-1}]	10.67	9.89	8.15	28.88	28.5	28.51	49.16	52.79	50.51
Maximum [µm s^{-1}]	17.89	17.92	17.93	40.78	40.59	40.51	71.23	83.41	85.73
**Strain**	***V. cholerae* Δ*pomAB* pAB-S26A**								
**Class**	**slow**			**medium**			**fast**		
**Speed range**	**4 – <18 µm s^{-1}**			**18 – <41 µm s^{-1}**			**41 µm s^{-1} or faster**		
**pH**	**7.0**	**8.0**	**9.0**	**7.0**	**8.0**	**9.0**	**7.0**	**8.0**	**9.0**
Number of tracks	459	363	565	184	238	78	7	49	6
Average [µm s^{-1}]	11.02	9.33	8.90	26.25	27.77	26.38	44.48	41.03	45.63
SD [µm s^{-1}]	3.10	3.55	2.96	6.14	6.46	6.17	3.75	4.94	5.38
Minimum [µm s^{-1}]	5.89	4.99	4.97	18.01	18.16	18.07	41.57	41.00	42.17
Median [µm s^{-1}]	10.26	7.79	7.95	25.11	27.08	24.07	42.52	46.27	43.49
Maximum [µm s^{-1}]	17.95	17.81	17.87	40.21	40.99	40.32	51.78	60.92	56.17
**Strain**	***V. cholerae* Δ*pomAB* pAB-S26T**								
**Class**	**slow**			**medium**			**fast**		
**Speed range**	**4 – <18 µm s^{-1}**			**18 – <41 µm s^{-1}**			**41 µm s^{-1} or faster**		
**pH**	**7.0**	**8.0**	**9.0**	**7.0**	**8.0**	**9.0**	**7.0**	**8.0**	**9.0**
Number of tracks	375	601	503	275	76	137	0	0	10
Average [µm s^{-1}]	13.52	7.96	9.26	21.63	25.16	26	0.00	0.00	45.56
SD [µm s^{-1}]	2.81	2.58	3.34	2.55	5.22	5.72	0.00	0.00	3.54
Minimum [µm s^{-1}]	7.51	4.38	4.33	18.03	18.10	18.05	0.00	0.00	41.01
Median [µm s^{-1}]	13.81	7.01	8.26	21.31	25.11	25.44	0.00	0.00	44.84
Maximum [µm s^{-1}]	17.97	17.92	17.93	29.56	36.62	39.25	0.00	0.00	51.53
