# Supplementary material for: Serine 26 in the PomB Subunit of the Flagellar Motor Is Essential for Hypermotility of Vibrio cholerae
Source: PLoS One. 2015 Apr 15;10(4):e0123518. doi: 10.1371/journal.pone.0123518 (PMC4398553; doi:10.1371/journal.pone.0123518)
Supplement: S4 Table — At pH 7.0, 8.0 and 9.0, the total numbers of tracks recorded with V. cholerae ΔpomAB expressing His6-PomA and wild type PomB-Strep were 649, 631 and 650, respectively. For His6-PomA together with PomB-S26A-Strep, the numbers of tracks were 640, 659 and 650, respectively, and 650, 650 and 650 for His6-PomA together with PomB-S26T-Strep. SD: Standard deviation. (PDF) [file pone.0123518.s004.pdf]

**Medium**	**LB with 171 mM K^+ added (LB-K^+)**								
**Strain**	***V. cholerae* Δ*pomAB* pAB**								
**Class**	**slow**			**medium**			**fast**		
**Speed range**	**4 – <18 µm s^{-1}**			**18 – <41 µm s^{-1}**			**41 µm s^{-1} or faster**		
**pH**	**7.0**	**8.0**	**9.0**	**7.0**	**8.0**	**9.0**	**7.0**	**8.0**	**9.0**
Number of tracks	289	275	488	317	227	98	43	129	64
Average [µm s^{-1}]	10.85	8.73	8.23	27.78	29.18	27.83	46.6	60.55	53.64
SD [µm s^{-1}]	3.59	3.34	2.7	6.23	6.47	6.37	5.31	10.82	9.19
Minimum [µm s^{-1}]	4.80	4.94	4.63	18.01	18.06	18.04	41.08	41.85	41.41
Median [µm s^{-1}]	9.87	7.31	7.22	27.29	28.93	27.68	45.62	58.85	51.23
Maximum [µm s^{-1}]	17.98	17.94	17.86	40.95	40.79	40.91	61.05	93.55	83.73
**Strain**	***V. cholerae* Δ*pomAB* pAB-S26A**								
**Class**	**slow**			**medium**			**fast**		
**Speed range**	**4 – <18 µm s^{-1}**			**18 – <41 µm s^{-1}**			**41 µm s^{-1} or faster**		
**pH**	**7.0**	**8.0**	**9.0**	**7.0**	**8.0**	**9.0**	**7.0**	**8.0**	**9.0**
Number of tracks	491	485	460	149	174	162	0	0	28
Average [µm s^{-1}]	8.73	11.98	10.03	24.94	23.13	26.41	0.00	0.00	49.04
SD [µm s^{-1}]	2.94	3.00	3.07	4.89	4.33	5.79	0.00	0.00	9.47
Minimum [µm s^{-1}]	5.50	5.22	5.57	18.11	18.05	18.04	0.00	0.00	41.11
Median [µm s^{-1}]	7.52	11.89	8.88	24.05	22.19	25.41	0.00	0.00	44.11
Maximum [µm s^{-1}]	17.89	17.92	17.95	39.70	37.64	40.51	0.00	0.00	71.24
**Strain**	***V. cholerae* Δ*pomAB* pAB-S26T**								
**Class**	**slow**			**medium**			**fast**		
**Speed range**	**4 – <18 µm s^{-1}**			**18 – <41 µm s^{-1}**			**41 µm s^{-1} or faster**		
**pH**	**7.0**	**8.0**	**9.0**	**7.0**	**8.0**	**9.0**	**7.0**	**8.0**	**9.0**
Number of tracks	519	472	239	131	135	374	0	43	37
Average [µm s^{-1}]	12.01	9.34	11.59	21.41	29.57	27.77	0.00	48.02	45.43
SD [µm s^{-1}]	3.14	2.91	3.17	3.29	7.14	5.76	0.00	4.58	3.52
Minimum [µm s^{-1}]	6.03	4.90	6.18	18.02	18.04	18.18	0.00	41.17	41.04
Median [µm s^{-1}]	11.75	8.49	11.16	20.29	31.32	27.47	0.00	47.44	44.83
Maximum [µm s^{-1}]	17.99	17.93	17.74	34.08	40.93	40.75	0.00	62.03	56.08
